# Early Postoperative PTH Determination as Predictor for Post-Thyroidectomy Hypocalcemia

**DOI:** 10.1055/a-2846-4602

**Published:** 2026-09-01

**Authors:** Ahmed Youssef, Amr Kholief, Austin Milton, Rusha Patel, Nilesh Vasan, Greg Krempl

**Affiliations:** 1 Department of Otolaryngology & Head and Neck Surgery, University of Oklahoma Health Sciences Center, Oklahoma City, Oklahoma, United States; 2 Department of Otolaryngology & Head and Neck Surgery, Alexandria University, Alexandria, Egypt

**Keywords:** thyroid, parathyroid, cancer, health planning guidelines

## Abstract

**Objective:**

Post-thyroidectomy hypocalcemia is a major complication following total thyroidectomy. This study aimed to evaluate early postoperative parathyroid hormone (PTH) measurement as a predictor of hypocalcemia after thyroidectomy and its impact on same-day discharge.

**Methods:**

This study combined a prospective IRB-approved correlation study of 60 patients who underwent total thyroidectomy with a meta-analysis of 5 observational studies.

**Results:**

Using Pearson Correlation Coefficient correlation, there was a significant positive correlation between PTH measured 1H after surgery with 4 H PTH (PCC = 0.787) and 4H Ionized Ca (PCC = 0.642). The sensitivity of early PTH measurement (cutoff < 14 pg/mL) for detecting hypocalcemia was 100%, with an AUC of 0.977 (95% CI: 0.565-1.0). Meta-analysis of previous studies confirmed a strong positive association between early PTH and late measurements.

**Conclusions:**

Early PTH measurement at 1 hour after surgery is a highly sensitive tool for detecting post-thyroidectomy hypocalcemia, which may help reduce hospital stay following thyroidectomy.

## Introduction

Total thyroidectomy is the standard surgical treatment for multiple benign and malignant conditions of the thyroid gland, including Graves' disease, compressive multinodular or substernal goiter, and thyroid cancer. Post-thyroidectomy hypocalcemia is one of the most common complications following total thyroidectomy. Transient and permanent hypocalcemia can be associated with serious neurological and cardiac symptoms, resulting in prolonged hospital stay and frequent rehospitalization. 1 2

There is no clear consensus on the appropriate timing for parathyroid hormone (PTH) assessment after a total thyroidectomy. There is also no universal standard for routine or targeted use of calcium/vitamin D supplements for patients after total thyroidectomy. The standard monitoring pathway for post-thyroidectomy hypocalcemia for many institutions historically includes post-operative PTH and calcium levels and observation thereafter. More recently, there has been increasing interest in and practice of outpatient total thyroidectomy procedures, leading to interest in early identification of patients at risk for postoperative hypocalcemia who may benefit from an inpatient stay.

In 2007, the Australian Endocrine Surgeons published guidelines for postoperative PTH measurement based on four studies combined data from 458 patients. 1 They recommended that all patients have PTH measured 4 hours postoperatively. They recommended that normal postoperative PTH levels are accurate in predicting calcium levels and allow for early discharge without supplementation; however, very low or undetectable PTH levels indicate the need for early initiation of treatment with oral calcium and calcitriol and monitoring of laboratory values; patients with intermediate levels should receive calcium supplementation and be monitored for hypocalcemia, as they may also require calcitriol. 1 The American Thyroid Association (ATA) similarly recommends post-operative PTH measurement to triage patients for hypocalcemia. In a position statement on postoperative hypocalcemia, the ATA points out that studies have examined post-operative PTH measurement windows of anywhere from 10 minutes to 1 day, with various cutoff levels being found to correlate with hypocalcemia. 2 3 4

Despite the lack of a universal standard, it has become accepted practice at our institution to discharge patients on the same day as their thyroidectomy with calcium supplementation if the 4-hour PTH is > 10 pg/dL. However, if surgery occurs later in the day, the 4-hour draw window, plus results time, can extend past reasonable discharge time frames and may necessitate an overnight admission. An earlier, accurate predictive measure of postoperative hypocalcemia may help avoid prolonged observation periods in otherwise discharge-ready patients. Instead of a 4-hour wait, we propose examining a 1-hour PTH level to predict postoperative hypocalcemia and assess readiness for discharge.

The aim of our study was to correlate blood levels of parathyroid hormone, (PTH), measured in the early postoperative period(1-hour), with blood levels of parathyroid hormone and ionized calcium measured after 4 hours in patients undergoing total thyroidectomy to determine if it is a predictor of hypocalcemia, and comparing this prospective study with the meta-analysis of previous data studies.

## Methods

An IRB-approved prospective study was conducted including patients scheduled for total or completion thyroidectomy at the University of Oklahoma Health Sciences Center. Exclusion criteria included patients with chronic kidney disease, or patients being on bisphosphonates or calcium regulating agents like thiazide diuretics or patients with history of parathyroid adenoma to minimize confounding so we can accurately assess the predictive value of early PTH in a physiologically stable population., or patients undergoing completion thyroidectomy with unknown preoperative PTH.

**Fig. 1:**
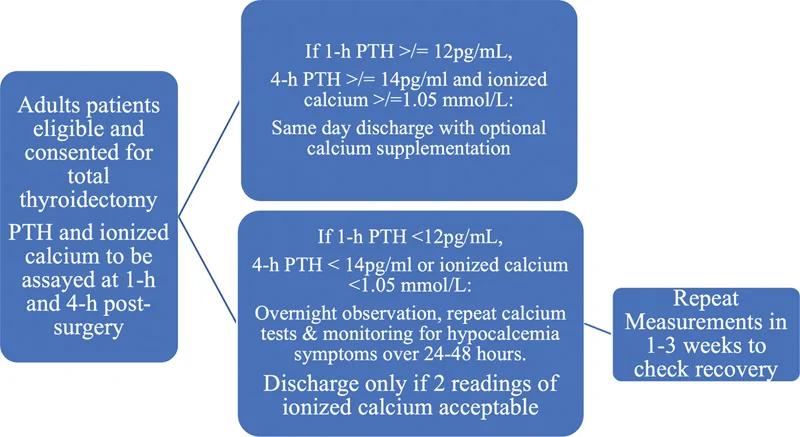
Protocol for monitoring hypocalcemia following total thyroidectomy.

As per our standard protocol ( Fig. 1 ), patients who underwent surgery were observed in the postoperative recovery area and a PTH and ionized calcium (iCa) were drawn 4 hours after surgery. A separate PTH level was drawn 1 hour after surgery per study protocol. The 4-hour PTH and iCal values were used to guide management, as is our standard practice:

Patients with a 4-hour postoperative PTH of >= 14 pg/mL and ionized calcium >= 1.05 mmol/L were discharged on the same day. Participants were informed of hypocalcemia symptoms and were given an emergency contact number to call if symptoms developed. Patients were also provided with calcium supplements but were instructed that supplementation was not required unless symptomatic. Patients with PTH or iCa levels below the thresholds listed above were admitted for observation with serum calcium measurements every 6 hours until two consecutive calcium levels were trending upward or acceptable for discharge. If measurements declined or patients developed hypocalcemia symptoms, replacement calcium and vitamin D were provided in the hospital and for home upon discharge. All patients were seen in clinic 1 week postoperatively for wound assessment, vocal cord examination, and repeat ionized calcium measurement ( Fig. 1 ).

At the time of analysis, 1-hour PTH was correlated with 4-hour PTH measurements. We also tested the sensitivity, specificity, and predictive value of the 1-hour measurements as a predictor for hypocalcemia. Linear regression analysis and Pearson correlation coefficient (PCC) were used to assess the correlation between PTH and calcium levels. Hypothesis testing of PCC = 0 was conducted.

For meta-analysis, A comprehensive literature search was conducted using the databases Medline, Embase (Ovid), Cinahl ('Cumulated Index to Nursing and Allied Health Care'; Ebsco collections) and the Cochrane Library, with results limited to papers published. 5 6 7 8 The search terms used were 'low calcium' OR 'hypocalcemia' AND 'thyroidectomy'. The full texts of all available relevant studies were reviewed independently by at least two authors (AELM, RB and VK). Data were independently extracted from papers that met the inclusion criteria using a piloted proforma. Any differences of opinion between authors regarding study eligibility or data extraction were discussed, and if dispute remained this was resolved by the primary author. Extracted data included study type, sample size, patient characteristics, definition(s) of hypocalcemia.

## Results

The prospective study included 60 participants: 20 males and 40 females. All patients underwent total thyroidectomy. Parathyroid gland preservation was performed in all cases. All thyroidectomy specimens were inspected by the surgical team before wound closure for any inadvertent parathyroid gland removal. None of these patients underwent parathyroid gland transplantation for this reason.

**Fig. 2:**
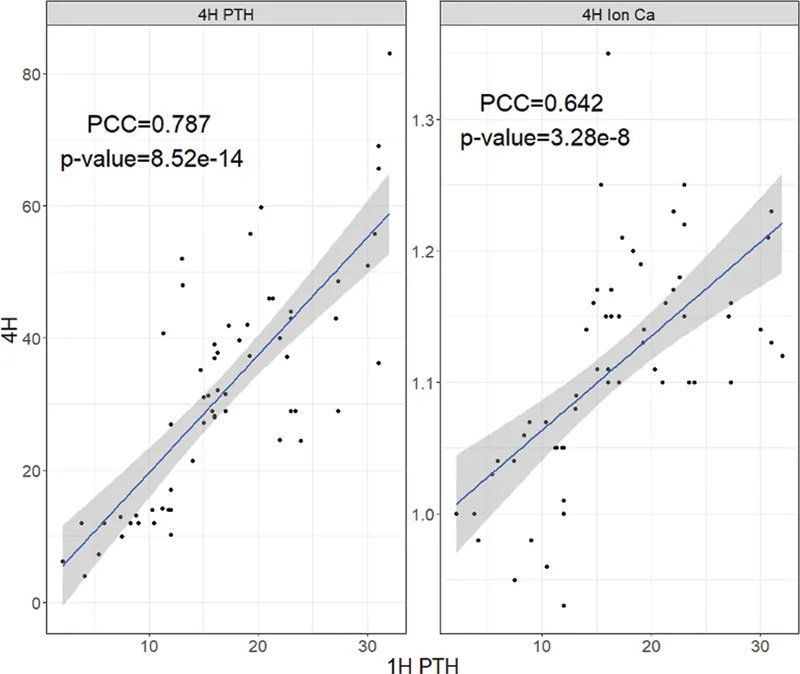
Linear regression analysis of the correlation between 1-hour PTH and 4-hour PTH and ionized calcium measurements.

There was a strong positive correlation between 1-hour PTH and 4-hour PTH /ionized calcium measurements. The Pearson correlation coefficient (PCC) between 1-hour and 4-hour PTH was 0.787 (p < 0.0001), and between 1-hour PTH and 4-hour ionized calcium was 0.642 (p < 0.0001) ( Fig. 2 ).

**Fig. 3:**
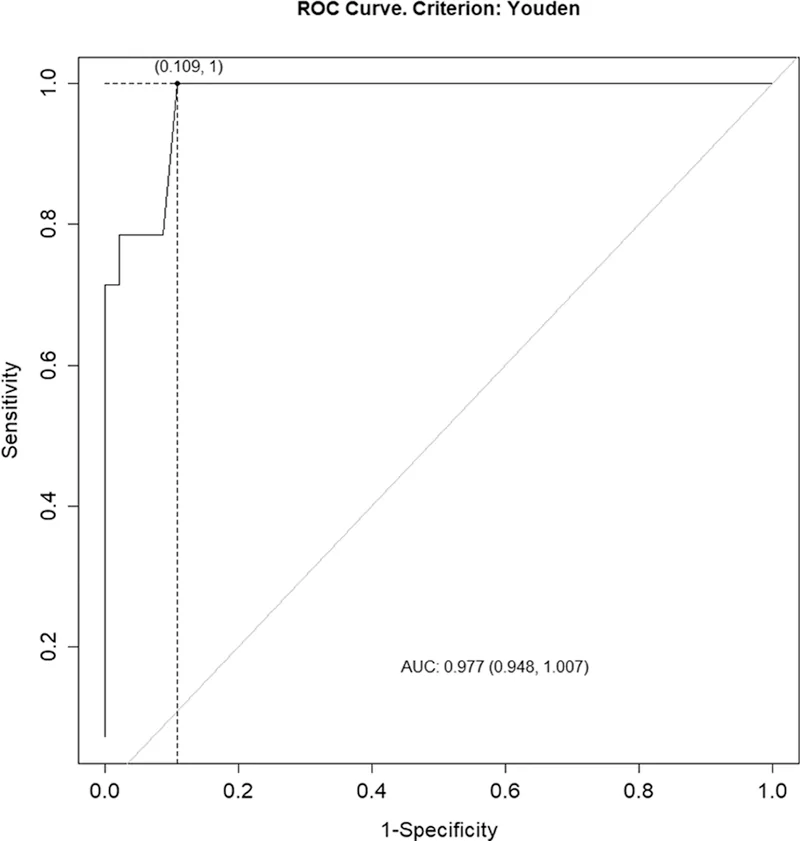
ROC analysis of 1-hour PTH cutoff value as a predictor of hypocalcemia.

A receiver operating characteristic (ROC) analysis was used to assess a 1-hour PTH cutoff value as a predictor of hypocalcemia. For a PTH cutoff of > 12 pg/mL, the sensitivity was 100%, specificity was 89.1%, positive predictive value was 73.7%, and negative predictive value was 100% ( Fig. 3 ).

**Fig. 4:**
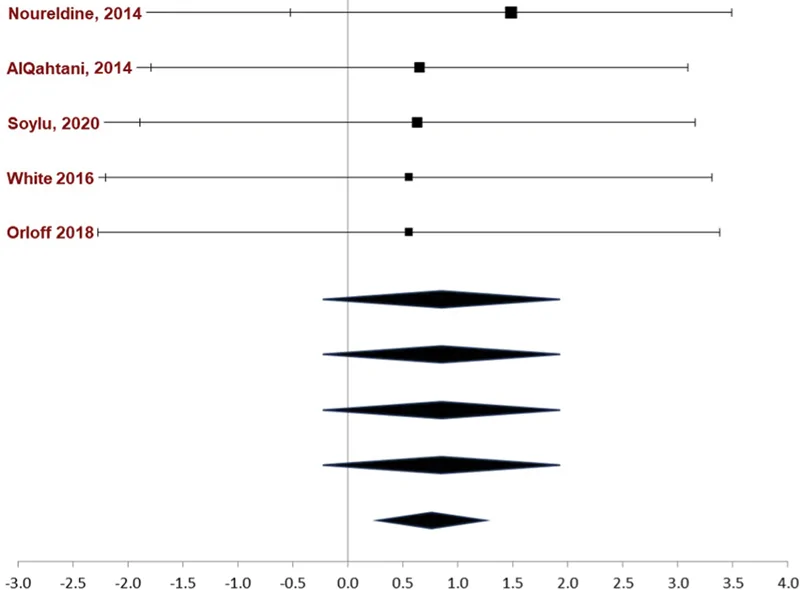
Forest plot of previous studies on early PTH measurement.

The meta-analysis of studies on hypocalcemia monitoring following thyroidectomy ( Table 1 ) showed a strong correlation between early and late PTH measurements and confirmed that early 1-hour PTH measurement had high sensitivity, specificity, and accuracy in predicting hypocalcemia. These findings are further supported by the meta-analysis by Orloff et al. 2 ( Fig. 4 )

**Table 1:** Summary of Studies on the Correlation between Early and Late Post-Thyroidectomy Serum PTH

Study	Year	Type of study	Dependent variable	Number of subjects	Correlation coefficient
White	2016	Prospective	PTH	196	0.522
Soylu	2020	Prospective	PTH	75	0.67
AlQahtani,	2014	Prospective	PTH	149	0.721
Noureldine,	2014	Prospective	PTH	304	0.62

## Discussion

Transient or permanent hypoparathyroidism as a potential sequela after total thyroidectomy remains an important post-surgical issue that can instigate or prolong hospital stay. Early identification of postoperative hypoparathyroidism can help anticipate early discharge and expedite care. To reduce costs and facilitate early hospital discharge, several studies have assessed the reliability of postoperative PTH measurement as a predictor of hypocalcemia, although cutoff levels vary among studies.

The present study adds to a body of literature supporting short-duration postoperative PTH measurement. 5 6 7 9 White et al.5 examined the utility of a 1-hour PTH level compared with a postoperative day 1 level. 5 They concluded that 1-hour PTH was equivalent to postoperative day 1 (POD1) PTH in predicting hypocalcemia, with a cutoff of 10 pg/dL. AlQahtani et al.7 prospectively examined 1-, 6-, and 24-hour postoperative PTH levels after thyroidectomy and found that a 1-hour PTH of 1.15 pmol/L was 100% specific and 89% sensitive in predicting postoperative hypocalcemia. 7 This data are consistent with the present results: we found that a 1-hour PTH that is ≥ 12 pg/mL is highly sensitive and specific (100%, 89.1%, respectively) and has a 100% negative predictive value in predicting postoperative hypocalcemia. Like prior studies, we found a strong correlation between 1-hour and 4-hour post-operative PTH levels. Although the question of early PTH in predicting hypocalcemia is not new, the present study contributes by corroborating prior findings and demonstrating reproducibility across institutions with different practice patterns. Although the present study is a single-institution prospective validation study, several limitations should be acknowledged. As a single-institution study with a limited sample size (n = 60), differences in assay techniques may cause variability when applying these cutoff values to other institutions. Additionally, cutoff values may not be generalizable to the general population, as the study excluded patients with a history of chronic kidney disease or calcium-altering conditions. Long-term clinical follow-up after protocol implementation was also lacking, which would strengthen the clinical argument for early PTH assessment. Finally, as mentioned previously, this study mirrors prior protocols and validates results from the authors mentioned. However, there is value in ensuring that the results of prior studies and those of the present study are in agreement. We hope this body of literature will be considered as a whole and can serve as a guideline for early PTH monitoring across different institutions and practice patterns. Future studies should focus on longer-term clinical follow-up for patients enrolled in an early PTH assay protocol following thyroid surgery.

## Conclusion

Early 1-hour PTH measurement is a highly sensitive tool for predicting hypocalcemia, with a negative predictive value of 100% in the present study. At our institution, 1-hour PTH measurement has been used to facilitate same-day discharge for many patients, with no readmission episodes. Implementation of these findings has made same-day total thyroidectomy safe and feasible for many patients.
